# Characterization of 19 Genes Encoding Membrane-Bound Fatty Acid Desaturases and their Expression Profiles in *Gossypium raimondii* Under Low Temperature

**DOI:** 10.1371/journal.pone.0123281

**Published:** 2015-04-20

**Authors:** Wei Liu, Wei Li, Qiuling He, Muhammad Khan Daud, Jinhong Chen, Shuijin Zhu

**Affiliations:** 1 Department of Agronomy, Zhejiang University, Hangzhou, 310058, China; 2 Department of Biotechnology and Genetic Engineering, Kohat University of Science and Technology, Kohat, 26000, Pakistan; 3 Jiangsu Collaborative Innovation Center for Modern Crop Production, Nanjing, 210095, China; National Key Laboratory of Crop Genetic Improvement, CHINA

## Abstract

To produce unsaturated fatty acids, membrane-bound fatty acid desaturases (FADs) can be exploited to introduce double bonds into the acyl chains of fatty acids. In this study, 19 membrane-bound FAD genes were identified in *Gossypium raimondii* through database searches and were classified into four different subfamilies based on phylogenetic analysis. All 19 membrane-bound FAD proteins shared three highly conserved histidine boxes, except for GrFAD2.1, which lost the third histidine box in the C-terminal region. In the *G*. *raimondii* genome, tandem duplication might have led to the increasing size of the FAD2 cluster in the Omega Desaturase subfamily, whereas segmental duplication appeared to be the dominant mechanism for the expansion of the Sphingolipid and Front-end Desaturase subfamilies. Gene expression analysis showed that seven membrane-bound FAD genes were significantly up-regulated and that five genes were greatly suppressed in *G*. *raimondii* leaves exposed to low temperature conditions.

## Introduction

Both saturated and unsaturated fatty acids are major components of membrane phospholipids in plants as well as triacylglycerols in seeds. Unsaturated fatty acids usually contain one or more double bonds in their acyl chains. The number and position of double bonds in fatty acids profoundly influence their physical and physiological properties [1,2]. The desaturation of fatty acids is catalyzed by a class of enzymes called fatty acid desaturases (FADs) [1,3]. The two major groups of fatty acid desaturases, soluble and membrane-bound, have been identified and have no evolutionary relationship with each other [4,5]. The soluble desaturases have two conserved histidine boxes and are represented by the plant stearoyl-ACP desaturase, which specifically desaturates stearoyl-ACP (18:0) to produce ACP-bound oleic acid (18:1) [6]. The membrane-bound desaturases contain three histidine boxes and are ubiquitous in prokaryotes and eukaryotes [4,5]. They comprise a highly diversified family that includes many different types of regioselectivities, such as Δ4, Δ5, Δ6, Δ7, Δ8, Δ9, Δ12 and Δ15 [5,7].

Most of the fatty acids residing in plant membranes are unsaturated. Their level of unsaturation is highly dependent upon the tolerance of a given plant for various environmental stresses, especially temperature stress [8,9,10]. Previous studies have revealed that genes encoding membrane-bound FAD proteins are crucial for the sustenance of plants faced with different environmental stresses. In rice, *OsFAD8* has been reported to have a functional role in stress tolerance at low temperatures [11]. *FAD2* and *FAD6* were found to be active in seedlings of *Arabidopsis* under salinity stress [12,13]. The *ads2* mutant *Arabidopsis* plants showed increased sensitivity to chilling and freezing temperatures [14], and the mutants of SLD genes also showed enhanced sensitivity to prolonged low-temperature exposure [15]. In tomato, *LeFAD3* over-expression enhanced the tolerance of tomato seedlings for salinity stress [16], whereas silencing the *LeFAD7* gene alleviated high-temperature stress [17]. In transgenic tobacco plants, over-expressing *FAD7* also showed enhanced cold tolerance [18], whereas antisense expression of the *Arabidopsis FAD7* reduced salt and drought tolerance [19]. In soybean, the expression of *FAD3* and *FAD7* was tightly regulated in response to cold temperature [20].

Cotton is the major source of natural fibers used in the textile industry. It is also a promising oilseed crop. Cotton is mostly grown in tropical and subtropical regions of the world, and its cultivation has been achieved even in relatively cold regions. Low temperature (under 15°C) can adversely affect plant development, resulting in poor germination and higher seedling mortality due to disease infection, which ultimately cause significant losses in yield [21]. Although the plant has been grown in cold areas, little is known about the molecular responses of cotton to low temperature. The Δ12 desaturases (FAD2) were extensively characterized in *Gossypium hirsutum* [21,22,23,24], and expression analysis suggested that FAD2 genes play a direct role in cotton adaptation to cold stress [21]. More recently, Δ15 fatty acid desaturases (FAD3 and FAD7/8) were identified in *Gossypium*, and one of the genes, termed *FAD7/8-1*, was dramatically induced during cold temperature treatment of *G*. *hirsutum* seedlings [25].

*Gossypium raimondii* is a diploid cotton species, whose progenitor is the putative contributor of the D subgenome to the economically important fiber-producing cotton species *G*. *hirsutum* and *G*. *barbadense* [26]. Sequencing of the *G*. *raimondii* genome has provided an opportunity for genome-wide analysis of all the genes belonging to specific gene families in cotton. In this paper, our main objectives were to identify membrane-bound FAD genes in *G*. *raimondii* through homology searches, to classify them into different subfamilies according to phylogenetic analysis, as well as to investigate their expression profiles in different tissues and under a cold stress regime. The results may provide information valuable for understanding the biological roles of membrane-bound FAD genes in the response of cotton to cold stress, and may also help cotton breeders improve the quality of cotton oil via molecular design breeding.

## Materials and Methods

### Database search and gene retrieval

The genome database (release v2.1) [27] of *G*. *raimondii* was downloaded from Phytozome (http://www.phytozome.net/). Seventeen membrane-bound fatty acid desaturases of *Arabidopsis* (S1 Table) [28,29] were obtained from the Arabidopsis Information Resource (TAIR release 10, http://www.arabidopsis.org/). To identify all candidate membrane-bound FAD genes of *G*. *raimondii*, these FAD protein sequences of *Arabidopsis* were employed as queries to search the *G*. *raimondii* genome database using BlastP and tBlastN programs with default parameters. Subsequently, the Pfam (http://pfam.sanger.ac.uk/search) [30] and SMART databases (http://smart.embl-heidelberg.de/) [31] were used to confirm each putative member of the FAD family. The theoretical Mw (molecular weight) and pI (isoelectric point) of the full-length protein were predicted using the ProtParam tool (http://web.expasy.org/protparam/).

### Multiple sequence alignment and phylogenetic analysis

Multiple sequence alignments of full-length protein sequences were performed using Clustal X version 2.0 [32] with default parameters. The Neighbor-Joining phylogenetic trees were constructed using MEGA 5.2 [33] with pairwise deletion option and poisson correction model. Bootstrap tests were carried out with 1000 replicates for statistical reliability.

### Gene structures, chromosomal locations and gene duplications

To illustrate exon-intron organization for an individual gene, the Gene Structure Display Server (GSDS, http://gsds1.cbi.pku.edu.cn/) [34] was employed to compare the predicted coding sequences (CDSs) with their corresponding genomic sequences.

The location data of all membrane-bound FAD genes were acquired from the genome annotation document. The chromosome location image of membrane-bound FAD genes was generated using MapInspect software according to their starting positions on the *G*. *raimondii* chromosomes [35,36].

Gene duplication of membrane-bound FAD genes in *G*. *raimondii* was defined according to (1) the length of aligned sequence cover was > 80% of the longer gene, (2) the identity of the aligned regions was > 80%, and (3) only one duplication event was counted for tightly linked genes [36,37,38]. With the chromosomal locations of membrane-bound FAD genes, two types of gene duplications were recognized (i.e., tandem and segmental duplications).

### Plant materials and low temperature stress treatment

All the plants of *G*. *raimondii* were grown in a temperature-controlled chamber at 28°C with a photoperiod of 16 hours light and 8 hours dark. After ten days, the leaves, stems, roots, and cotyledons of some seedlings were sampled to analyze tissue-specific expression. To examine the expression patterns of membrane-bound FAD genes under low temperature stress, the plant leaves of the remaining seedlings treated at 10°C in the temperature-controlled chamber were harvested at 0, 3, 6, and 12 hours, which represented normal plants, slight stress, moderate stress, and severe stress, respectively. All collected samples were immediately frozen in liquid nitrogen and stored at -80°C. Three biological replicates were conducted per sample.

### RNA isolation and quantitative real-time RT-PCR (qRT-PCR)

Total RNA was extracted from all samples using the EASYspin Plus Total RNA Extraction Kit (Aidlab, Beijing, China), and first-strand cDNAs were synthesized with the PrimeScript 1st Strand cDNA Synthesis Kit (TakaRa, Dalian, China) according to the manufacturer’s protocols. For quantitative real-time RT-PCR (qRT-PCR) assay, gene-specific primers were designed for the membrane-bound FAD genes according to their CDSs (S2 Table). The qRT-PCR was performed with the SYBR *Premix Ex Taq* (TakaRa, Dalian, China) in the BioRad CFX96 Real-time PCR System following the manufacturer’s instructions. The cotton *UBQ7* gene was used as an internal reference for all the qRT-PCR analyses. Each sample was performed in three biological replicates. The relative expression levels were calculated according to the 2^-ΔΔCT^ method [39]. The expression profiles were clustered using the Cluster 3.0 software [40].

## Results

### Identification of membrane-bound FAD genes in *G*. *raimondii*

The candidate membrane-bound FAD genes were identified from the *G*. *raimondii* genome using the BlastP and tBlastN programs with the query sequences of *Arabidopsis* membrane-bound FAD genes. The retrieved sequences were submitted to the Pfam and SMART databases to confirm the presence of conserved domains (Pfam: PF00487). As a result, 19 non-redundant membrane-bound FAD genes were confirmed in *G*. *raimondii*. For comparative analysis, the membrane-bound FAD genes in rice (S1 Table) were also identified from the Rice Genome Annotation Project Database (RGAP release 7, http://rice.plantbiology.msu.edu/index.shtml) following the same strategy. All identified FAD genes were named according to their orthology with reported counterparts in *Arabidopsis*. Detailed information about the 19 membrane-bound FAD genes in *G*. *raimondii* is provided in Table 1. The protein sequences encoded by these 19 FAD genes varied in length from 292 amino acids for GrFAD2.1 to 477 amino acids for GrFAD8.2, with an average of approximately 397 amino acids. The predicted molecular weight (Mw) of these proteins ranged from 33.44 kDa to 55.30 kDa, and the theoretical isoelectric point (pI) ranged from 6.95 to 9.61.

**Table 1:** Basic information of the membrane-bound FAD genes in *G*. *raimondii*.

No.	Gene name	Locus ID	Protein length	Mw (kDa)	pI
1	*GrFAD2*.*1*	Gorai.013G248700	292	33.44	9.61
2	*GrFAD2*.*2*	Gorai.013G248800	383	43.86	8.95
3	*GrFAD2*.*3*	Gorai.002G156500	383	44.31	8.94
4	*GrFAD2*.*4*	Gorai.002G156600	377	43.70	8.95
5	*GrFAD2*.*5*	Gorai.007G362300	384	44.30	9.05
6	*GrFAD6*	Gorai.013G216300	442	51.34	9.17
7	*GrFAD3*.*1*	Gorai.001G116500	376	43.59	8.68
8	*GrFAD3*.*2*	Gorai.006G106100	388	45.03	9.05
9	*GrFAD7*	Gorai.011G289600	450	51.17	8.91
10	*GrFAD8*.*1*	Gorai.012G115000	446	50.72	8.52
11	*GrFAD8*.*2*	Gorai.002G262800	477	55.30	7.78
12	*GrDSD1*	Gorai.010G153100	324	37.70	6.95
13	*GrDSD2*	Gorai.011G026500	331	38.64	8.36
14	*GrSLD1*	Gorai.004G286900	447	51.24	8.61
15	*GrSLD2*	Gorai.009G044600	447	51.20	8.68
16	*GrSLD3*	Gorai.008G122500	325	37.03	7.27
17	*GrSLD4*	Gorai.007G104200	447	51.29	8.77
18	*GrSLD5*	Gorai.001G158100	447	51.18	8.55
19	*GrFAD5*	Gorai.009G128400	386	44.32	9.44

### Phylogenetic analysis of membrane-bound FAD genes

**Fig 1:**
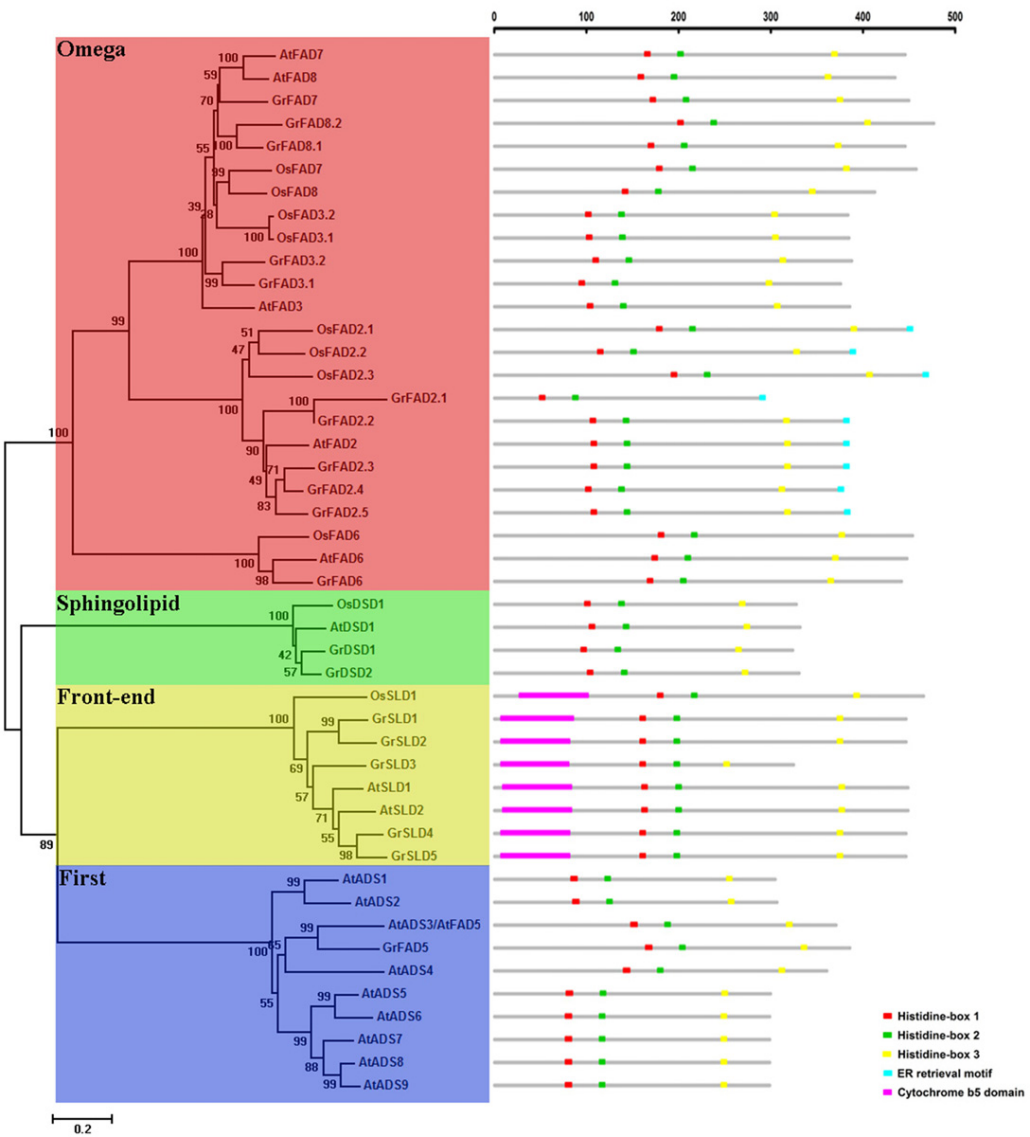
Phylogenetic relationships and motif compositions of membrane-bound FAD genes from *G*. *raimondii*, *Arabidopsis* and rice. Unrooted phylogenetic tree (left panel): Four subfamilies marked with different color backgrounds are labeled as Omega, Sphingolipid, Front-end and First. Motif compositions (right panel): Protein sequences are indicated by thick gray lines, and the conserved motifs are represented by different colored boxes. The length (amino acids) of the protein and motif can be estimated using the scale bar at the top.

To evaluate the phylogenetic relationships of membrane-bound FAD genes in different species, an unrooted phylogenetic tree was constructed according to the alignments of full-length protein sequences of membrane-bound FADs in *G*. *raimondii*, *Arabidopsis*, and rice. In previous reports, membrane-bound desaturases from eukaryotic genomes were divided into four functional subfamilies: First Desaturase, Omega Desaturase, Front-end Desaturase, and Sphingolipid Desaturase [7]. As shown in the phylogenetic tree (Fig 1), all of the membrane-bound desaturases used in this study fell into these four subfamilies.

The First Desaturase subfamily included Δ7 desaturases and Δ9 desaturases, encoded by ADS genes, which generally introduced the first double bond into the saturated acyl chain [7,41]. There were nine members of the subfamily in *Arabidopsis*, but only one gene was found in *G*.*raimondii*, which was a homolog of *AtADS3* (also termed *AtFAD5*).

The Omega Desaturase subfamily contained Δ12 desaturases and Δ15 desaturases, which introduce a double bond between an existing double bond and the acyl end [3]. The Δ12 desaturases encoded by FAD2 and FAD6 were frequently called ω6 desaturases [42,43], and the Δ15 desaturases encoded by FAD3, FAD7 and FAD8 were also called ω3 desaturases [44,45,46]. In the phylogenetic tree, FAD2 and FAD6 genes were grouped in separate branches. *G*. *raimondii*, *Arabidopsis* and rice had one FAD6 gene each, but the number of FAD2 genes was diverse, with an expanded number (up to five) in *G*. *raimondii*, which was greater than that in *Arabidopsis* (one) and rice (three). FAD3, FAD7, and FAD8 formed the other cluster, which contained three FAD genes from *Arabidopsis*, four from rice, and five from *G*. *raimondii*.

The Front-end Desaturase subfamily was comprised of sphingolipid Δ8 desaturases, which were encoded by SLD genes [7,15]. Five SLD genes were found in *G*. *raimondii*, two SLD genes in *Arabidopsis*, and one SLD gene in rice.

The last group was the Sphingolipid Desaturase subfamily, which was represented by sphingolipid Δ4 desaturases [7,47]. The group contained one gene from *Arabidopsis*, one gene from rice, and two genes from *G*. *raimondii*.

Interestingly, the number of members identified in *G*. *raimondii* was greater than that in *Arabidopsis* and rice in three of four subfamilies (Table 2). This result suggested that the membrane-bound FAD genes in the *G*. *raimondii* genome might have undergone species-specific expansion over the course of evolution.

**Table 2:** The number of membrane-bound FAD genes of *Arabidopsis*, rice and *G*. *raimondii* in four subfamilies.

Subfamily	*Arabidopsis*	Rice	*G*. *raimondii*
First Desaturase	9	0	1
Omega Desaturase	5	8	11
Front-end Desaturase	2	1	5
Sphingolipid Desaturase	1	1	2
Total	17	10	19

### Conserved motifs in membrane-bound FAD genes

The membrane-bound desaturases shared three highly conserved histidine boxes, which were thought to be involved in the formation of the active site of each desaturase [1,48]. All of the membrane-bound FAD proteins analyzed in this study contained these three histidine boxes, except for GrFAD2.1, which lost the third histidine box in the C-terminal region (Fig 1, S3 Table). Additionally, the relative positions of the three histidine boxes in the protein sequences were similar among desaturases. The first and second histidine boxes were located near each other, with only 31 or 32 amino acid residues between them. The intervening length in the First and Omega Desaturase subfamilies was 31 amino acid residues, and there were 32 amino acid residues in the Front-end and Sphingolipid Desaturase subfamilies. The third histidine box was positioned in the C-terminal region of the proteins. The number of amino acid residues between the second and third histidine boxes was different among subfamilies, but in each subfamily or cluster, the number was almost identical. For example, there were 127 amino acid residues in the First Desaturase subfamily and 161 or 162 amino acid residues in the FAD3/FAD7/FAD8 cluster of the Omega Desaturase subfamily.

To further confirm the conservation of amino acid residues in the histidine boxes, the sequence logos of the three histidine boxes in each subfamily were generated using the WebLogo program (S1 Fig). As previously reported [49,50,51], it was observed that the first residue in the third histidine box was glutamine rather than histidine in the Front-end Desaturase subfamily. Apart from this divergence, the remaining histidines were strongly conserved among subfamilies. However, the other amino acids in the histidine boxes differed greatly. Remarkably, there were four amino acid residues between the histidines in the first histidine-box of the First Desaturase subfamily, but only three in the other three subfamilies.

FAD2, known as the ER-localized membrane-bound FAD, contained an ER (endoplasmic reticulum) retrieval motif consisting of Φ-X-X-K/R/D/E-Φ (Φ are large hydrophobic amino acid residues such as F/Y/W/I/L/V) at the C-terminus [52]. Expectedly, all five FAD2 proteins identified in *G*. *raimondii* had the ER retrieval motif (Fig 1). The motif was YHNKF in GrFAD2.1, YRNKF in GrFAD2.2, FRNKL in GrFAD2.3, FRNKL in GrFAD2.4, and FRNKI in GrFAD2.5, respectively. SLD, which functions as a sphingolipid Δ8 desaturase, was characterized by the presence of an N-terminal cytochrome *b*_*5*_ domain [53]. Through searches in Pfam database, it was found that all five SLD genes in *G*. *raimondii* contained the cytochrome *b*_*5*_ domain at the N-terminus (Fig 1).

### Chromosomal locations and structure of membrane-bound FAD genes

**Fig 2:**
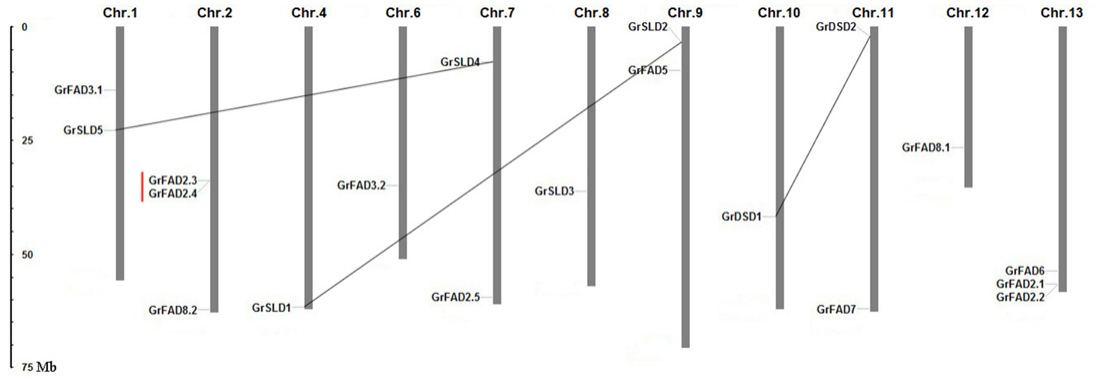
Chromosomal localization of membrane-bound FAD genes in *G*. *raimondii*. Chromosome numbers are indicated above each vertical bar. The scale is in megabases (Mb). The black lines connecting the genes indicate segmental duplications, and tandem duplications are marked by red lines.

The 19 membrane-bound FAD genes were mapped to the 11 chromosomes in *G*. *raimondii* (Fig 2). They were distributed unevenly among the chromosomes. Chromosomes 2 and 13 contained three membrane-bound FAD genes each and chromosomes 1, 7, 9, and 11 contained two genes each, while only a single FAD gene was localized on each of the chromosomes 4, 6, 8, 10, and 12. There were no FAD genes located on chromosomes 3 and 5. Four duplicated gene pairs, i.e., *GrFAD2*.*3*/*GrFAD2*.*4*, *GrDSD1*/*GrDSD2*, *GrSLD1*/*GrSLD2*, and *GrSLD4*/*GrSLD5*, were found in the *G*. *raimondii* genome. According to the chromosomal distribution of the membrane-bound FAD genes, three duplication events were assigned to the segmental duplication. *GrFAD2*.*3* and *GrFAD2*.*4*, which were positioned adjacently on chromosome 2 with no intervening genes, were involved in a tandem duplication event.

**Fig 3:**
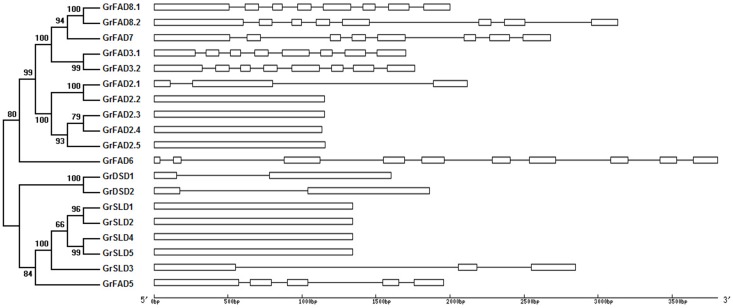
Phylogenetic relationship and gene structure of membrane-bound FAD genes in *G*. *raimondii*. Exons are represented by white boxes and introns by black lines.

A separate phylogenetic tree was generated using the protein sequences of all membrane-bound FAD genes identified in *G*. *raimondii* and the exon-intron structures of these genes were compared (Fig 3). All members of the FAD3/FAD7/FAD8 cluster contained eight exons. Their conserved gene structure supported their close evolutionary relationship. Most of the FAD2 genes, including the duplicated genes *GrFAD2*.*3* and *GrFAD2*.*4*, had only one exon, with the exception of *GrFAD2*.*1*, which contained three exons. For SLD, the genes of two duplicated pairs, *GrSLD1*/*GrSLD2* and *GrSLD4*/*GrSLD5*, had the same gene structures and contained one exon. *GrDSD1* and *GrDSD2*, another duplicated gene pair, contained two exons. However, *GrSLD3* had three exons, *GrFAD5* had five exons, and *GrFAD6* contained up to ten exons.

### Expression of the membrane-bound FAD genes in different tissues

**Fig 4:**
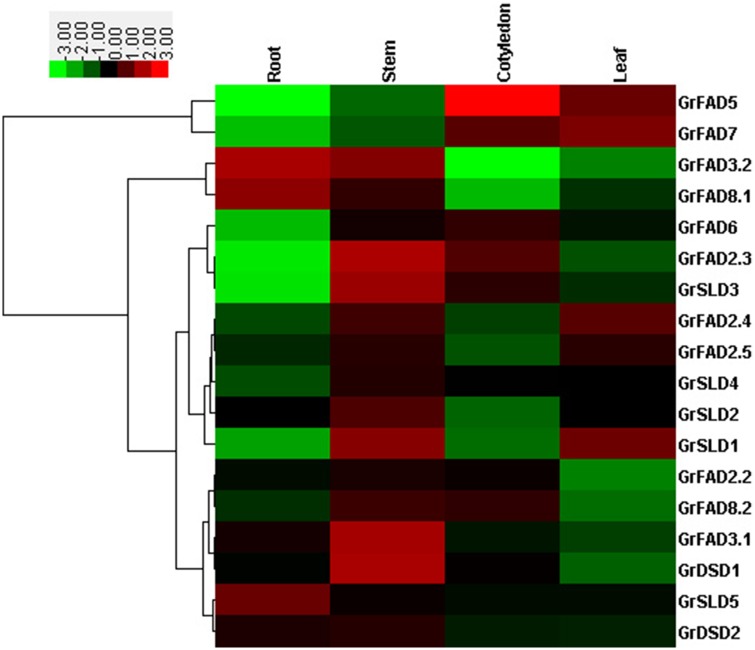
Expression patterns of membrane-bound FAD genes in four representative tissues of *G*. *raimondii* seedlings. The color scale represents the gene expression intensity, green indicating low levels of transcript abundance and red indicating high transcript abundance.

To investigate the expression profiles of membrane-bound FAD genes in different tissues of *G*. *raimondii*, qRT-PCR analysis was performed to examine the gene expression levels in the roots, stems, cotyledons, and leaves of 10-day-old seedlings. Because no transcripts could be detected for *GrFAD2*.*1* in the four representative tissues using up to five gene-specific primers in reverse transcription (RT)-PCR analysis (data not shown), this gene was not included in the qRT-PCR analysis. As shown in Fig 4, the expression patterns of the 18 membrane-bound FAD genes varied significantly in the tissues analyzed in this study. *GrFAD3*.*2*, *GrFAD8*.*1*, and *GrSLD5* were expressed at high levels in roots. *GrFAD2*.*3*, *GrSLD3*, *GrSLD2*, *GrSLD1*, *GrFAD3*.*1*, and *GrDSD1* shared high expression levels in young stems. *GrFAD5* and *GrFAD6* displayed the highest transcript abundance in cotyledons. And *GrFAD7* and *GrFAD2*.*4* were predominantly expressed in leaves. These results demonstrated that the majority of these membrane-bound FAD genes exhibited tissue-specific expression patterns, which was consistent with membrane-bound FAD genes in *Arabidopsis* and soybean, which also showed specific spatial expression patterns [28].

Furthermore, the tissue-specific expression patterns of the genes involved in duplication events were compared. Although the genes in all four duplicated gene pairs shared high sequence similarity and the same gene structure, the gene expression patterns were highly diverse. For instance, *GrFAD2*.*3* showed preferential expression in the stem and cotyledon, but that in the root and leaf was limited. And *GrFAD2*.*4*, which was involved in a duplication event with *GrFAD2*.*3*, showed preferential expression in the stem and leaf.

### Expression patterns of membrane-bound FAD genes under low temperature

**Fig 5:**
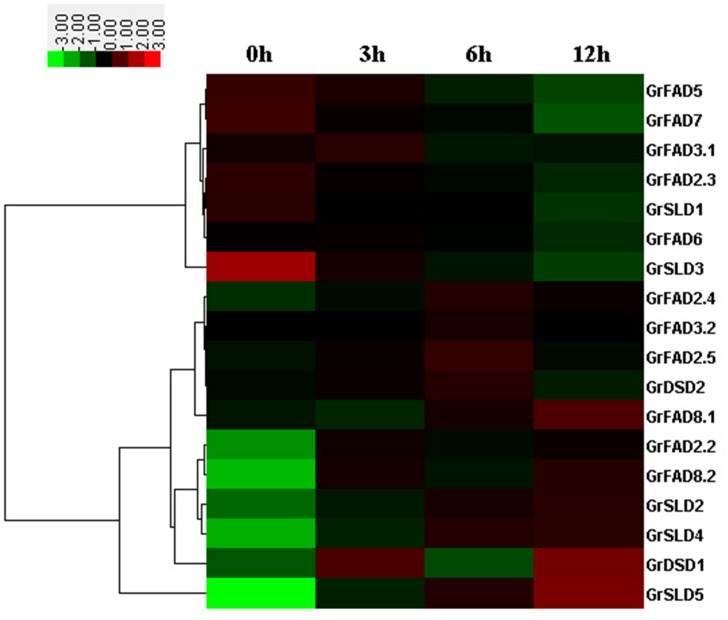
Expression profiling of membrane-bound FAD genes in leaves under 10°C treatment. The color scale represents the gene expression intensity, green indicating low levels of transcript abundance and red indicating high transcript abundance.

The expression patterns of the *G*. *raimondii* membrane-bound FAD genes in the leaves of 10-day-old seedlings under low temperature stress (10°C) were investigated in this study, and all the gene expression levels responsive to slight (3 hours), moderate (6 hours), and severe (12 hours) cold stresses were compared with those of normal plants as shown in Fig 5, with the exception of *GrFAD2*.*1*. Out of the 18 membrane-bound FAD genes, seven genes, i.e., *GrFAD8*.*1*, *GrFAD2*.*2*, *GrFAD8*.*2*, *GrSLD2*, *GrSLD4*, *GrDSD1*, and *GrSLD5*, showed a marked increase in transcript level when treated with cold stress. Among them, the expression levels of *GrFAD8*.*1* and *GrSLD5* were higher at 12 hours after cold treatment, and *GrSLD2* and *GrSLD4* almost reached their highest levels at 6 hours after cold treatment, whereas *GrFAD2*.*2*, *GrFAD8*.*2*, and *GrDSD1* were expressed highly at 3 hours of cold treatment. Additionally, the expression levels of 5 other genes, i.e., *GrFAD2*.*4*, *GrFAD3*.*1*, *GrFAD3*.*2*, *GrFAD2*.*5*, and *GrDSD2*, were slightly up-regulated in response to cold stress. In contrast, the five genes *GrFAD5*, *GrFAD7*, *GrFAD2*.*3*, *GrSLD1*, and *GrSLD3* were significantly down-regulated after long periods of cold stress treatment, and one gene, *GrFAD6*, was suppressed slightly during treatment with cold stress. There were also some differences in expression patterns between duplicated genes under low temperature.

## Discussion

FAD genes encode the enzymes that catalyze the desaturation of fatty acids, which affect the oxidative stability and nutritional value of seed storage oils [54]. Many studies have indicated that modifying the activity of FAD genes could create transgenic soybean lines with improved seed oil quality through altering relative amounts of fatty acids [54,55,56,57]. Cotton is also a significant oilseed crop, and cottonseeds are an important source of livestock feed, foodstuff and oil [58]. FAD2 genes have already been genetically manipulated for cottonseed oil improvement [59,60,61]. However, only several FAD genes encoding the Δ12 and Δ15 desaturases have been characterized in cotton [22,23,24,25]. In this study, a comprehensive set of 19 non-redundant membrane-bound fatty acid desaturases was identified from the available genome sequences of *G*. *raimondii*. Undoubtedly, the 19 membrane-bound FAD genes identified in the diploid cotton will provide candidate genes for the gene engineering of fatty acid biosynthesis in cotton.

The FAD genes were named based on their orthologous genes in *Arabidopsis*. However, following this nomenclature, it was difficult to distinguish *FAD7* and *FAD8*, due to their high degree of homology. It has been demonstrated that *FAD7* was highly expressed at high temperatures [11,62] and that the transcript level of *FAD8* increased at low temperatures [11,62,63,64]. Therefore, according to the expression patterns of FAD genes under low temperature stress analyzed in this study, *GrFAD7* and two isoforms of *GrFAD8* (*GrFAD8*.*1* and *GrFAD8*.*2*) were designated. *GrFAD7* was suppressed at low temperature, whereas *GrFAD8*.*1* was consistently induced under long duration cold exposure and *GrFAD8*.*2* was rapidly induced at 3 hours under low temperatures.

Analysis of protein structure showed that all of the 19 membrane-bound desaturases in *G*. *raimondii*, except for GrFAD2.1, had the three highly conserved histidine boxes, which contained strongly conserved histidine residues. GrFAD2.1 contained the first and second histidine boxes in the N-terminal region, but lost the third histidine box. Moreover, the gene structure of *GrFAD2*.*1* was also different from other FAD2 genes. By using five gene-specific primers, *GrFAD2*.*1* was not found to be expressed in this study. These observations suggested that *GrFAD2*.*1* might not be a functional gene. Considering the adjacent location of *GrFAD2*.*1* to *GrFAD2*.*2* on chromosome 13, it could be deduced that *GrFAD2*.*1* might have undergone significant functional divergence or even have become a pseudogene after originating from an ancient tandem duplication event of the FAD2 gene in the *G*. *raimondii* genome.

Gene duplications play a significant role in the expansion of gene families in the genome [65,66]. The total number of membrane-bound FAD genes identified in *G*. *raimondii* was much greater than that in *Arabidopsis* and rice. Phylogenetic analysis of membrane-bound desaturases in *G*. *raimondii*, *Arabidopsis* and rice indicated that all subfamilies except for the First Desaturase subfamily contained more gene members in *G*. *raimondii* than in *Arabidopsis* and rice. The increased number of members of the *G*. *raimondii* membrane-bound FAD gene family belonging to the three subfamilies suggested that they might have undergone species-specific expansion during the process of evolution. In this study, the gene duplication events, including tandem and segmental duplications, were investigated to elucidate the expansion mechanism of the membrane-bound FAD gene family in *G*. *raimondii*. Four duplicated gene pairs, including eight genes out of the 19 membrane-bound FAD genes, were identified. Among them, one tandem duplicated gene pair, *GrFAD2*.*3*/*GrFAD2*.*4*, belonged to FAD2 cluster of the Omega Desaturase subfamily. A segmental duplicated gene pair, *GrDSD1*/*GrDSD2*, belonged to the Sphingolipid Desaturase subfamily. And the remaining two segmental duplicated gene pairs, *GrSLD1*/*GrSLD2* and *GrSLD4*/*GrSLD5*, belonged to the Front-end Desaturase subfamily. These results showed that the tandem duplication might contribute to the increasing size of the FAD2 cluster and that the expansion of the Sphingolipid and Front-end Desaturase subfamilies was due to the segmental duplication.

Duplicated genes may experience three outcomes, i.e., non-functionalization (loss of original functions), neo-functionalization (acquisition of novel functions), or sub-functionalization (partition of original functions), during the process of evolution [67]. Gene expression profiles can provide useful clues for understanding the function of these genes. According to the experiments characterizing tissue-specific expression or response to low temperature performed in this paper, the expression patterns of members in the four duplicated gene pairs were significantly diverse, which indicated that the functions of the duplicated genes were strongly differentiated after duplications. The fate of the duplicated genes could be described as neo-functionalization.

Low temperature is one of the serious environmental stresses affecting cotton development and production. Previous studies revealed that FAD2 and FAD7/8 genes participated in cotton adaptation to cold stress [21,25], and accumulating evidence has indicated that many members of the membrane-bound FAD gene family were involved in the response to cold stress in numerous plant species [9,10,11,14,15,18,20]. In this study, the expression patterns of the *G*. *raimondii* membrane-bound FAD genes in leaves under low temperature were investigated at the whole family level. As a result, *GrFAD8*.*1*, *GrFAD2*.*2*, *GrFAD8*.*2*, *GrSLD2*, *GrSLD4*, *GrDSD1* and *GrSLD5* were found to be significantly up-regulated in response to cold stress, which suggested that these genes might be required to maintain appropriate levels of related unsaturated fatty acids in cotton plants under low temperature conditions. Conversely, *GrFAD5*, *GrFAD7*, *GrFAD2*.*3*, *GrSLD1* and *GrSLD3* were heavily down-regulated after long periods of cold stress treatment. In the previous study, two FAD2 genes, designated *FAD2-3* and *FAD2-4*, were found to be induced in upland cotton (*G*. *hirsutum*) under cold stress [21]. In this study, four functional isoforms of FAD2 genes were identified in *G*. *raimondii*, of which *GrFAD2*.*2* was induced under low temperature, *GrFAD2*.*4* and *GrFAD2*.*5* were slightly up-regulated in response to cold, and *GrFAD2*.*3* was suppressed under low temperature. These results suggested that specific isoforms of FAD2 genes might play a vital role in cotton response to cold stress.

SLD genes encoding the sphingolipid Δ8 desaturases have been well studied in *Arabidopsis*, and deficiency of the genes resulted in enhanced sensitivity to prolonged low-temperature exposure [15]. Here, *GrSLD2*, *GrSLD4*, and *GrSLD5* were highly expressed after long periods of cold treatment, whereas *GrSLD1* and *GrSLD3* were suppressed under low temperatures, suggesting that SLD genes are critical for cotton response to cold stress.

## Supporting Information

S1 Fig Sequence logos of the three histidine boxes in four subfamilies. The height of the letter designating the amino acid residue at each position represents the degree of conservation. The numbers on the x-axis represent the residue positions within the boxes. The y-axis represents the information content measured in bits. Note that all protein sequences in each subfamily were included in the analysis, with the exception of *GrFAD2*.*1*, which was excluded from the Omega Desaturase subfamily.
(TIF)

S1 Table The membrane-bound fatty acid desaturases in *Arabidopsis* and rice. (XLSX)

S2 Table PCR primers used in this study. (XLSX)

S3 Table The conserved histidine boxes of membrane-bound FAD proteins in *G*. *raimondii*, *Arabidopsis* and rice. (XLSX)
