# Supplementary figures and images for: Characterization of 19 Genes Encoding Membrane-Bound Fatty Acid Desaturases and their Expression Profiles in Gossypium raimondii Under Low Temperature
Source: PLoS One. 2015 Apr 20;10(4):e0123281. doi: 10.1371/journal.pone.0123281 (PMC4404247; doi:10.1371/journal.pone.0123281)

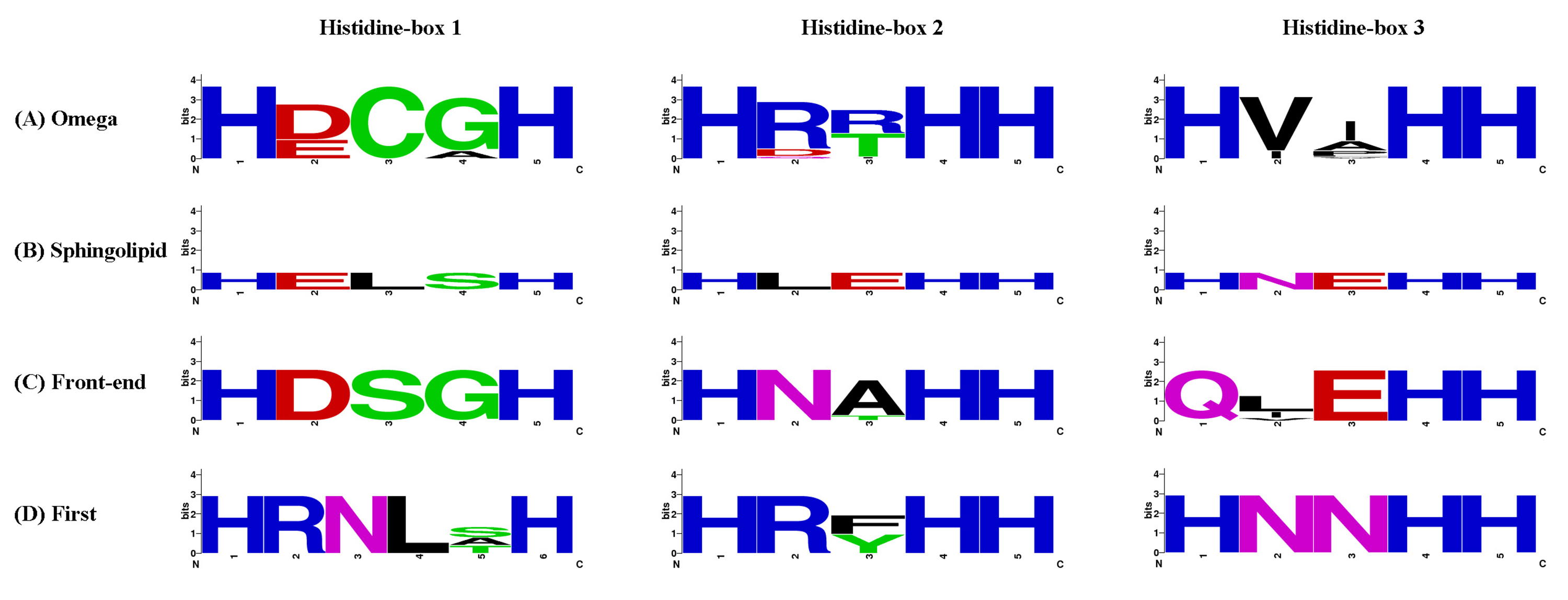

Supplement: S1 Fig — The height of the letter designating the amino acid residue at each position represents the degree of conservation. The numbers on the x-axis represent the residue positions within the boxes. The y-axis represents the information content measured in bits. Note that all protein sequences in each subfamily were included in the analysis, with the exception of GrFAD2.1, which was excluded from the Omega Desaturase subfamily. (TIF) [file pone.0123281.s001.tif]
